# Evaluation of organizational climate factors on tax administration enterprise resource planning (ERP) system

**DOI:** 10.1016/j.heliyon.2022.e09642

**Published:** 2022-06-04

**Authors:** Godwin Banafo Akrong, Yunfei Shao, Ebenezer Owusu

**Affiliations:** a School of Management and Economics, University of Electronic Science and Technology of China, Chengdu, 611731, China; b Center of West African Studies, University of Electronic Science and Technology of China, Chengdu, 611731, China; c Department of Computer Science, University of Ghana, Ghana

**Keywords:** Enterprise resource planning (ERP) systems, DeLone & McLean IS success model, Information systems, Organizational climate, Structural equation modeling

## Abstract

Tax collection is an essential activity to boost the economy of all countries. Larger businesses and governments are increasingly relying on Enterprise Resource Planning (ERP) systems, which are designed to enhance the collection of revenues among other things. However, the implementation of an ERP system often affects the organizational climate by changing the manner businesses are conducted from the past both internally and externally. These changes have the tendency to impact the actions of workers throughout the transition process. Nevertheless, organization climate which is an essential variable to measure the success of ERPs is mostly underutilized. Thus in this study, we proposed an information system (IS) success model that integrates organizational climate variables namely, role clarity, teamwork and support, and, training and learning into the DeLone and McLean model to evaluate the success of a tax ERP system. The proposed model was based on a quantitative and a mixed-method case study (MM-CS). Data was gathered from a top company with many branches in Ghana through interviews, observation, focus groups, and questionnaires. Partial least squares structural equation modeling was used to examine the 555 data collected from the questionnaire. The result of the study shows that the organizational climate variables (training & learning, teamwork & support, and role clarity) were statistically significant in determining the success of a tax ERP system. Training & learning and teamwork & support also had a positive impact on service quality, user satisfaction, and individual impact.

## 1 Introduction

Income generation via tax is critical for government growth in underdeveloped nations. However, without accurate digital data, a good tax return is unattainable, since the majority of developing nations are progressively transitioning away from manual recordkeeping (Sigala et al., 2020). Larger businesses and governments are increasingly turning to Enterprise Resource Planning (ERP) solutions, which are designed to integrate seamlessly into an organization's environment over time (Chofreh et al., 2020). ERP systems are one of the key technology instruments being utilized by governments worldwide to boost productivity, reduce costs, and enhance citizen services (Chatzoglou et al., 2016).

Most companies' senior management believes that integrating an ERP system into their company would result in a dramatic improvement in the organization's performance and that of the workers who work with the installed system (Qutaishat et al., 2012; Uwizeyemungu and Raymond, 2010). According to Trucco and Corsi (2014), ERP systems have not always gained substantial recognitions in organizations as expected because there exists some lower degree of user acceptability. Nonetheless, researchers and information system developers have not given up on developing new solutions that will improve user acceptability, ERP system use, and methods for determining an ERP system's success (Abdinnour and Saeed, 2015; Alhirz and Sajeev, 2015; Rezvani et al., 2017; Zabukovsek and Bobek, 2013). These solutions paved the way for the adoption of ERP systems in tax administration (Moreno-Jiménez et al., 2013). ERPs are described as computerized management systems that facilitate the storing and processing of data and assist in the effective administration of administrative operations (Uddin et al., 2020). As a result, they may be seen as instruments for rationalization, as they integrate the operational activities of corporate functions into a single system. Additionally, ERP systems provide an option that enables businesses to modify the way they manage their workers' expertise and management.

However, the change process in an organization presents numerous challenges that must be addressed, particularly when an ERP system is implemented in the day-to-day activities of employees. Organizational changes are sometimes adequately planned for and, at other times, foisted (Pasmore and Woodman, 2017). When adequately planned for, organizations can anticipate and implement measures to control change (Al-Haddad and Kotnour, 2015; Greenwood and Hinings, 1996; Král and Králová, 2016). Controlling unforeseen challenges, on the other hand, is a little more precarious in the case of an organization imposing the change (Czekster et al., 2019). The changes that occur in an organization as a result of the implementation of an ERP, whether planned or imposed, tend to affect the activities of employees during the change process (Cui and Jiao, 2019), and changes in an organization are frequently influenced by organizational climate. As the effect, of organizational climate, maybe a result of the introduction of new technologies, social values, economic variables, and government actions (e.g., tax law changes) (Buchanan, 2019; Poole and Van de Ven, 2004; Mutonyi et al., 2020).

Thus, one could argue that organizational climate affects the implementation, use, user satisfaction, and overall success measurement of an ERP. The success of an ERP system can be determined by examining the information quality, service quality, and system quality of the implemented system, as well as the system's use and user satisfaction (DeLone and McLean, 1992, 2003). According to Furnham and Goodstein (1997), organizational climate encompasses a variety of dimensions, including role clarity, career development, respect, communication, reward systems, planning and decision-making, innovation, relationships, teamwork and support, service quality, conflict management, commitment and morale, training and learning, and direction. Dinibutun et al. (2020) identified additional dimensions of organizational climate as managerial competence, balanced workload, task clarity, cohesion, ethics, and participation. Additional organizational climate dimensions identified were role, job, organization, and supervision (Li et al., 2020). Nkasu (2020) also did extensive work on the effect of key success factors (CSFs) on ERP system adoption. According to the findings, only ten of the different CSFs found in the study had the most significant impact on the effective deployment of an ERP system, and this includes user training and education, clear goals and objectives, and learning competence. Interestingly, teamwork and composition were ranked 15th on the list. Again, according to Vargas and Comuzzi (2020), user training and education were critical elements in the implementation of an ERP system. Barth and Koch (2019) indicates that external support and the ERP team must be given enough attention for an installed ERP system to be effective. Thus, the outcome of the study shows that when companies attempt to quantify the success of an implemented ERP, organizational climate factors that have been experimentally shown to account for crucial ERP success should be included in the IS success model. This should be done with a particular emphasis on the influence of organizational climate on the DeLone and McLean (1992, 2003) proposed IS quality dimensions, as well as on usage, user satisfaction, and net benefit (individual impact and organizational impact).

Though organizational climate variables throughout time have been recognized as key success indicators for the adoption of an ERP system, previous research has not utilized them in tax ERPs success models. As a result, we sought to address this issue by adding these organizational climate variables (role clarity, teamwork and support, and, training and learning) into the DeLone and McLean IS success model. The study's findings indicated that these factors provide a substantial amount of value to the model for measuring tax ERP. Additionally, we evaluated the variables that influence the use of a tax ERP system, user satisfaction, information quality, service quality, and system quality, as well as the aspects that impact the system's overall success.

The study makes four significant contributions to the literature. Firstly, this study proposed that factors relating to organizational climates, such as training & learning, teamwork & support, and role clarity, should be incorporated in the IS success model when evaluating the overall success of a tax ERP system. Secondly, the study evaluates the proposed model in a tax ERP system. Thirdly, we examined the relationship between the various IS quality constructs and found that they are significantly related. Additionally, the influence of organizational climate on the quality constructs of IS, their usage, user satisfaction, individual impact, and the organizational impact was examined and found that training and learning, and teamwork and support contrary to popular opinions do not influence the use of a tax ERP.

The remaining section of this work is organized as follows: Section 2 discusses the theoretical foundations, organizational climate, the ERP system, and the related model. The proposed model and hypotheses are discussed in Section 3. Section 4 discusses the empirical approach used to construct the model and the process by which the model was validated. Section 5 provides a sneaky summary of the results, and Section 6 discusses them. The final conclusion of the study is drawn in section 7.

## 2 Literature review

### 2.1 Organizational climate

The organizational climate is a critical factor in the success of the vast majority of businesses. This is because it can assist businesses in achieving their objectives and has an effect on many areas of the workplace. The organizational climate acts as an unwritten rule book, defining what is and is not acceptable behavior (Kılıç and Altuntaş, 2019). Schneider et al. (2013) assert that organizational climate refers to workers' common views of and interpretations of the rules, practices, and procedures they encounter. Also, employees are recognized to often ascribe meaning to the rules that tie them, and these views eventually coalesce into a climate (Ali et al., 2018; Huang et al., 2017). This indicates that organizational climate may be a useful variable to investigate and study since it may provide employees a feeling of control over their actions. Especially at an era when the majority of companies in developing countries are striving to embrace and implement enterprise resource planning (ERP) systems that have gained popularity in developed countries.

According to the reviewed literature, various climate dimensions exist, including role clarity, career development, respect, communication, reward system, planning and decision-making, innovation, relationships, teamwork and support, service quality, conflict management, commitment and morale, training and learning, and direction (Furnham and Goodstein, 1997). Alternatively, Datta and Singh (2018) defined five aspects of organizational climate: Professionalism, Organization & Workgroup, Facilitation & Support by Leaders, Cohesion, Clarity & Objectivity of System and Job Challenge, Variety & Feedback. Further study, identified the following climate dimensions as critical to organizational climate: training, motivation, supervision, safety, and resources (Escamilla-Fajardo et al., 2021), customer service, reputation, facilitation of work, participation, communication, team building, decision making, and compensation (Mohamed and Gaballah, 2018). These dimensions have been identified as the primary focus of the majority of climate studies when investigating the relationships between organizational climate and other variables (Hashim et al., 2015; Jaafari et al., 2012). Organizational climate is flexible and varies throughout various companies owing to the behavior, rules, procedures, or practices in place, thus there is no uniform set of characteristics (Berberoglu, 2018).

### 2.2 Enterprise resource planning

ERP is an important operational mechanism in day-to-day corporate operations, and the advantages gained from ERP systems differ from firm to firm. However, recent theoretical advances indicate that certain common advantages endure. This emanates from IT infrastructure, followed by operational, organizational, and managerial advantages (Weli, 2019). Businesses nowadays appear to gain from data reporting as exposure for firms and employees that effectively utilize ERP systems. Obtaining real-time data from companies and maintaining regulatory compliance allows for this (Fadelelmoula, 2018). Many companies adopt ERP systems for this reason (Sriram et al., 2020). An ERP system also improves a company's competitiveness and performance (Kulikov et al., 2020; Mahraz et al., 2020). This saves money, eliminates silos, and simplifies processes (Badewi et al., 2018; Trinoverly et al., 2018). Process enhancements and KPI analysis will help organizations increase productivity (AboAbdo et al., 2019).

These key metrics are achieved by ERP users who are satisfied with the outcomes (Al-Fawaz et al., 2010; Bramantoro, 2018). This improves customer experience, consumer connections, and builds new operating models. Companies gain a competitive advantage by abandoning old systems and depending on change management. Businesses implementing ERP systems will not gain market advantages if end-users cannot embrace the newest technology (Kanellou and Spathis, 2013; Mahraz et al., 2020). Despite the benefits of an ERP, Prasetyo et al. (2019) report a significant failure rate. A rigorous framework to assess the effectiveness of such systems in developing countries is therefore required.

### 2.3 Related models

**Figure 1:**
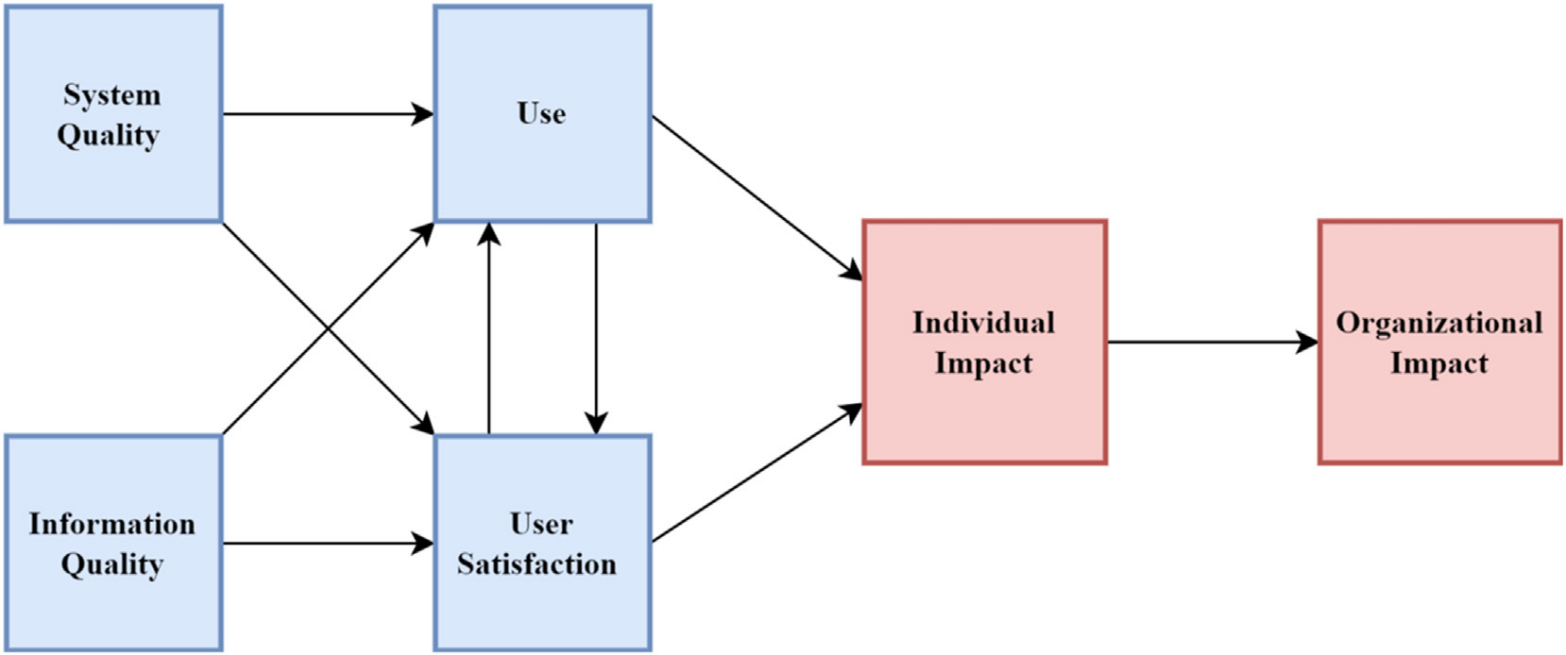
DeLone and McLean IS success model (1992).

DeLone and McLean (1992) identified six variables of information system (IS) success; *system quality, information system*, *use*, *user satisfaction*, *individual impact, and organizational impact.* However, these variables were not dependent on success measures. Seddon and Kiew (1996) further modified the *use* construct in the DeLone and McLean (1992) model by arguing that people are more interested in *usefulness*. The proposal of Seddon and Kiew (1996) was similar to that of the Technology Acceptance Model (TAM) (Davis, 1989). Seddon and Kiew (1996) further argued that in systems that *use* is voluntary, the variable *use* is an appropriate measure, but in a situation where *use* is mandatory, *usefulness* becomes a better measure of IS success. DeLone and McLean (2003) disagreed with Seddon and Kiew (1996) stance and acknowledged that even in mandatory systems, there is still a level of variability to use and called for *use* to be maintained as presented in Figure 1.

**Figure 2:**
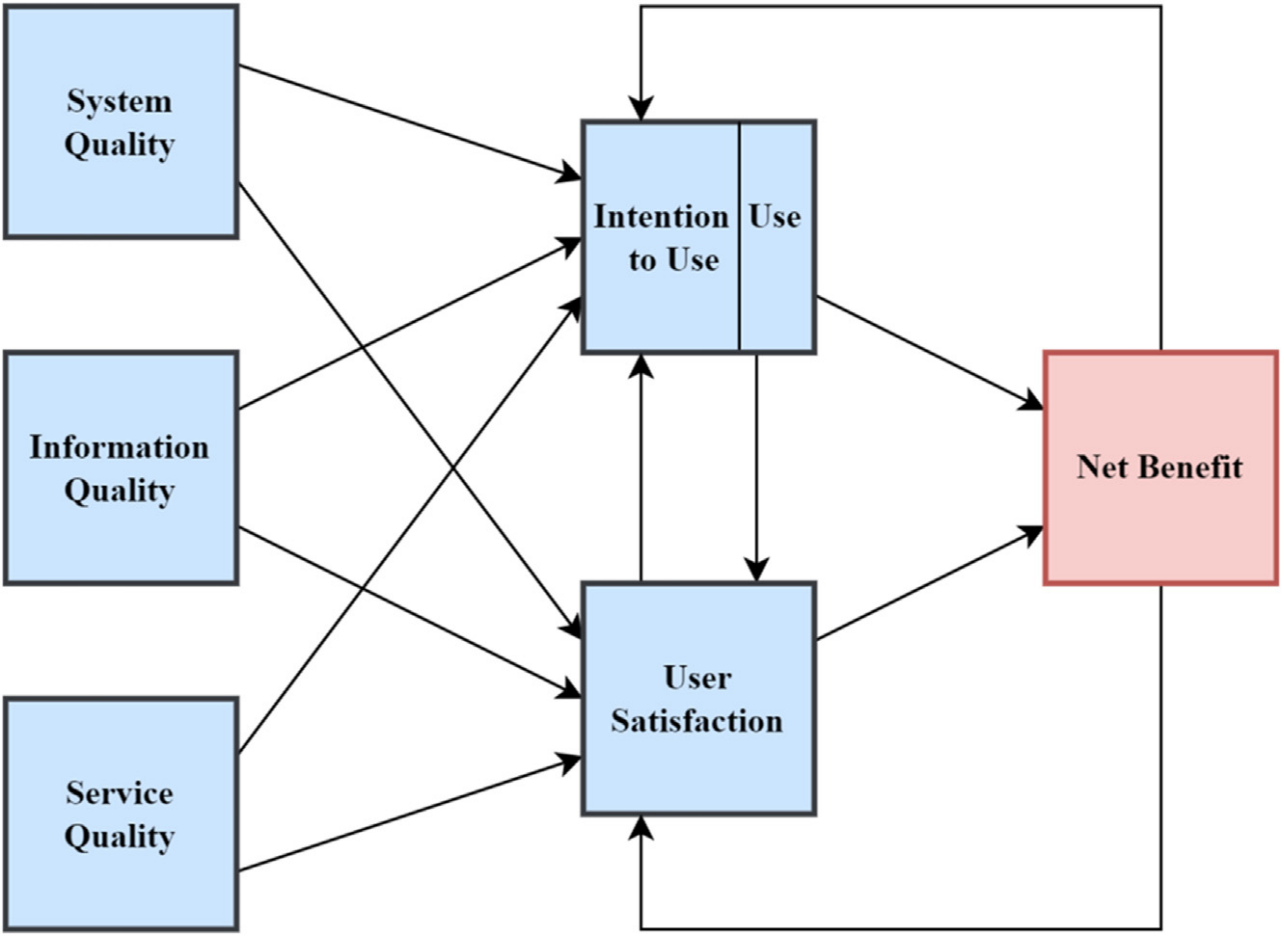
Updated DeLone and McLean IS success model (2003).

DeLone and McLean (2003) updated their model, considering the recommendations of Pitt et al. (1995) to include *service quality* as a construct. *Individual impact* and *organizational impact* were merged into a *net benefit* to help measure the benefits at multiple levels. The introduction of *net benefit* was a result of a recommendation from Myers et al. (1997) and Seddon and Kiew (1996). The next modification was about the *use* construct. DeLone and McLean (2003) argued that *use* precedes *user satisfaction* in the process sense, but a positive experience with *use* leads to higher *user satisfaction* in a causal sense. They also proposed that an increase in *user satisfaction* leads to a higher *intention to use*, which subsequently affect *use*. The updated model consisted of six variables. *Intention to use* and *use* replaced the initial *use* construct and the *individual impact*, and the *organizational impact* was merged into one construct net benefit, as presented in Figure 2.

## 3 The proposed model

**Figure 3:**
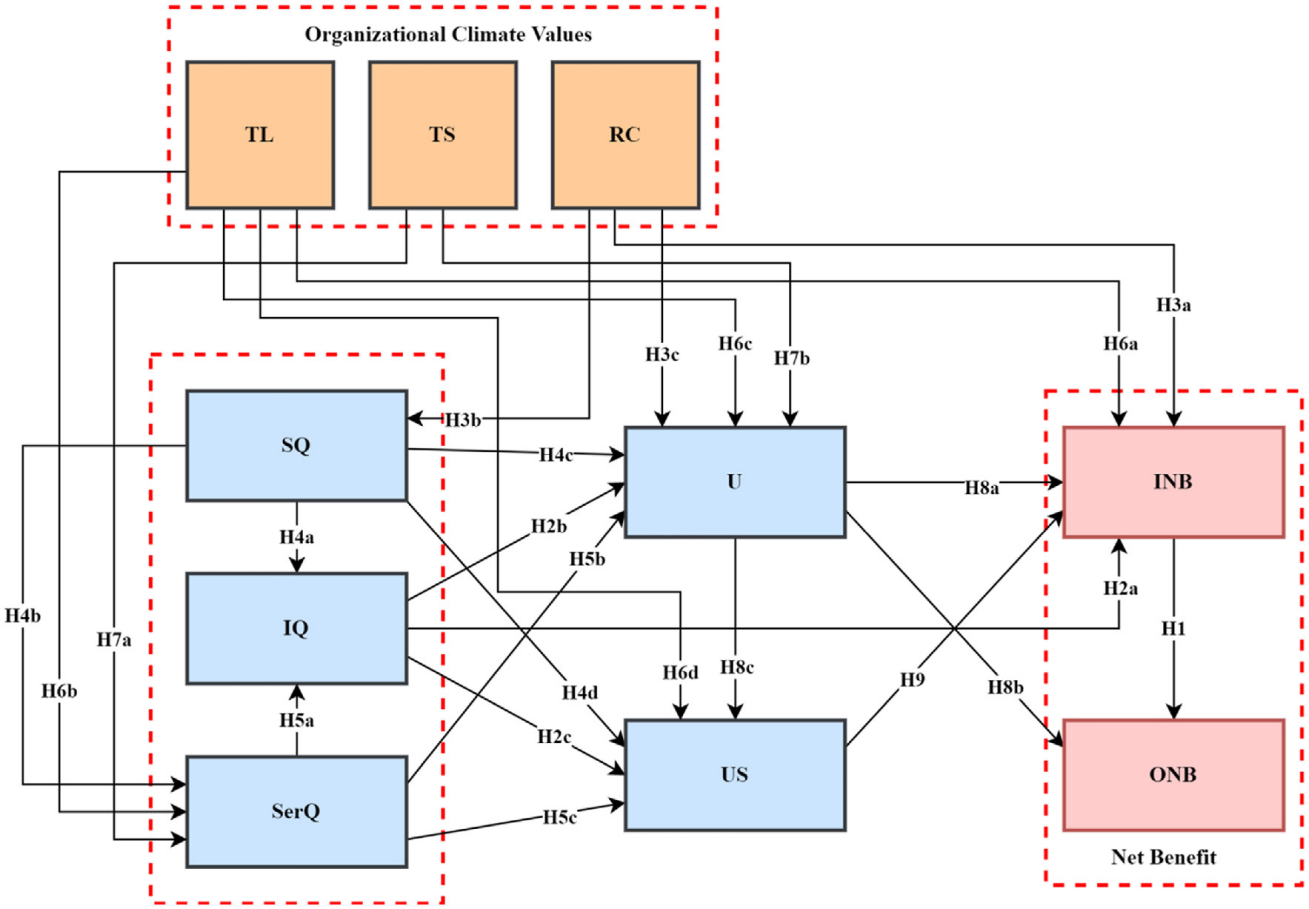
Conceptual research model. Organizational climate values: Training & Learning (TL); Teamwork & Support (TS); Role Clarity (RC) DeLone and McLean Model: System Quality (SQ); Information Quality (IQ); Service Quality (SerQ); Use (U); User Satisfaction (US); Individual Impact (INB); Organizational Impact (ONB).

Figure 3 shows the framework of the study which advances on the DeLone and McLean (1992, 2003) model.

We updated the DeLone and McLean IS success model for this study by integrating organizational climate values and assessing their influence on the variables that contribute to the success and use of the adapted ERPx (for confidentiality reasons, the assessed ERP is termed ERPx). This was the conclusion of our prior exploratory qualitative research in the three study regions. The exploratory qualitative research showed that *organizational climate* factors among employees had evolved once the ERPx system was fully implemented. These climate variables have been identified as having a major effect on the ERP system's adoption and success (Alsharari et al., 2020; Bhatt et al., 2021; Nkasu, 2020). Indicating that when ERP systems/modules are deployed, a well-designed *training & learning* process assists users in learning how to utilize the system while becoming satisfied (Gavali and Halder, 2020). *Teamwork & support*, as well as *role clarity*, were identified as important determinants in ERP system success, as well as their use and satisfaction (Vargas and Comuzzi, 2020; Yadav and Joseph, 2020). As a result, these organizational climate variables must be fully scrutinized and incorporated in the evaluation of an ERPs success and use. As a result, these organizational climate variables must be thoroughly examined and factored into the assessment of an ERP's success and implementation.

*Training & Learning* is a term that refers to the coaching given, the development of skills, and the process through which users master the system's usage. *Teamwork and support* relate to an ERP user's ability to get quick and efficient help from teammates, service providers, and the information technology department. The degree to which goals and objectives, priorities, responsibilities, and information about the majority of departments are obvious is quantified by the term "*role clarity*." The proposed model evaluated the effect of three distinct organizational climate values (*Training and Learning, Teamwork and Support,* and *Role Clarity*) on *system quality, information quality, service quality, use, user satisfaction,* and *net benefit (individual impact* and *organizational impact).*

As a result, we proposed that organizational climate be fully included as a construct in the DeLone and McLean IS success model when evaluating the success of a tax ERP system, as it has been presented that organizational climate influences the implementation and assessment of an IS success. Tax corporations who have adopted such ERPs and are attempting to quantify their success on both an *individual* and *organizational level* will lose out if these *organizational climate* values are not included.

The revised model developed by DeLone and McLean (2003) combines *actual usage* and *intention to use*. The proposed model retained just the *use* construct since the ERPx system utilized in the research was obligatory for users. By excluding the *intention to use* construct from the proposed model, the impact of *use* on *user satisfaction* is assessed independently of the impact of *user satisfaction* on *use*. *Individual impact* and *organizational impact* both result in a *net benefit*, according to DeLone and McLean (1992). This concept was included in the proposed model to allow the study of success on both the individual and organizational levels, but the study put a higher premium on the individual level. Additionally, the proposed model included the influence of *service quality* and *system quality* on *information quality*, allowing for quantification of the impact of *information quality* on *individual impact* (IBN). Secondly, we suggested that system quality has an impact on service quality. Thirdly, the impact of the three quality constructs (*Information quality, System quality, and Service quality*) on *use* and *user satisfaction* was measured. Finally, a relationship was proposed between *individual impact* (INB) and *organizational impact* (ONB).

### 3.1 Hypotheses development

#### 3.1.1 Individual impact (IBN) and organizational impact (ONB)

The individual impact is the degree to which an ERP system impacts users' expertise and efficiency while executing activities. Individual productivity, job effectiveness, task performance, and job simplicity influence organizational impact items including overall success and quality enhancement (A. H. Aldholay et al., 2018; Krasniqi et al., 2019). DeLone and McLean (2016) assert that end-users may identify such effects once the technology is adopted. Individual impact positively influences organizational impact (Roky and Meriouh, 2015); hence, the following hypothesis is proposed:

H1 The individual impact from Tax ERP systems is positively related to organizational impact.

#### 3.1.2 Information quality(IQ)

The desired qualities of an ERP system's output are its information quality. This includes, but is not limited to, Tax ERP system availability, relevance, timeliness, security, and dependability. According to Chen et al. (2019) study, information quality has a significant impact on job effectiveness and individual productivity. Information quality is also important in creating a favorable attitude about the advantages of using particular information systems (Mellouli et al., 2020). It is often seen as a crucial antecedent of user satisfaction because it influences users' attitudes about ERP system satisfaction, which in turn drives their perceptions about the system's satisfaction and flexibility (Sebetci, 2018). Several studies have emphasized the effect of information quality on user satisfaction, indicating that information quality is the main element driving satisfaction with information system use (Al-Okaily et al., 2021; Arain et al., 2019; Mohammadi, 2015; Wang and Teo, 2020). Moreover, there is enough evidence to prove that there is also a significant relationship between information quality and the use of an information system (Aldholay et al., 2019; Chen, 2020; Stefanovic et al., 2016). Therefore, the following hypotheses (H2a-c) are advanced:

H2a The information quality of a Tax ERP is positively associated with individual impact.

H2b The information quality of a Tax ERP is positively related to the use of an ERP system.

H2c The information quality of a Tax ERP is positively related to the user satisfaction of an ERP system.

#### 3.1.3 Role clarity (RC)

ERP users need to understand their roles and responsibilities to perform their work effectively. It is quantified by examining the clarity of goals and objectives, priorities, duties, and knowledge of the majority of departments (Furnham and Goodstein, 1997; Cäker and Siverbo, 2018). These approaches have a substantial influence on the work productivity of information system (IS) users, enable them to do their everyday tasks effectively, and encourage job simplification (Pahi et al., 2020). This shows how role clarity affects individual impact. Additional research corroborates this relationship, indicating that role clarity improves job satisfaction and also improves individual performance (Kalliath et al., 2012; Orgambídez and Almeida, 2020). The rationale that an IS user uses an implemented Tax ERP is because their role concerning the system is defined. Role clarity also has a significant effect on system quality because it enables an individual to readily utilize the system to accomplish particular tasks and appreciate system features such as flexibility, timeliness, and speed. We, therefore, advanced the following hypotheses:

H3a Role clarity is positively related to individual impact.

H3b Role clarity is positively related to the system quality of a Tax ERP.

H3c Role clarity is positively related associated with Use of a Tax ERP.

#### 3.1.4 System quality (SQ)

System quality relates to how user-friendly and hassle-free systems are. System quality includes ease of use, system reliability, system learning, system responsiveness, and system flexibility (Petter et al., 2008). The simplicity of utilizing a Tax ERP system makes it feasible for the information provided by the system to be relevant and trustworthy (Santa et al., 2019). This is because users seldom encounter problems. Admittedly, the system is simple to use and comprehend. Navigation and screen design may help enhance users' desire to utilize the system (Al-rawahna et al., 2019). As a result, a Tax ERP users strive for system quality compatibility (Chen et al., 2019). Other studies show a significant relationship between system quality and user satisfaction (Alkraiji, 2020) and system quality and use (Mishra and Geleta, 2020). The service quality of an ERP system is also influenced by system quality characteristics (Ariana et al., 2020). Because when a system encourages learning, speed, integration, and customization, it tends to impact service quality such as interpersonal quality and intrinsic quality. Therefore, we proposed the following hypothesize (H4a-d).

H4a The system quality of a Tax ERP is positively related to the information quality of a Tax ERP system.

H4b The system quality of a Tax ERP is positively related to the service quality of a Tax ERP system.

H4c The system quality of a Tax ERP is positively associated with the use of a Tax ERP system.

H4d The system quality of a Tax ERP is positively associated with user satisfaction.

#### 3.1.5 Service quality (SerQ)

Service quality is related to responsiveness, assurance, technological expertise, employee assistance, and dependability. The most often used metric is SERVQUAL, and studies indicate that active technical staff contributes to future use and user satisfaction (DeLone and McLean, 2003; Irawan and Syah, 2017). Assurance and support tend to influence information quality (Arias and Maçada, 2018). In terms of providing relevant, timely, and reliable data, users of a Tax ERP systems need to be guaranteed of data security and availability (Ameen et al., 2019; Mukamurenzi et al., 2019). Sachan et al. (2018) and Veeramootoo et al. (2018) established the relationship between service quality and user satisfaction. Some other studies indicated a positive relationship between service quality and system use (Kumar et al., 2020; Nishant et al., 2019). Based on these studies, we propose the following hypotheses.

H5a Service quality of a Tax ERP is positively associated with the information quality of a Tax ERP system.

H5b Service quality of a Tax ERP is positively associated with the use of a Tax ERP system.

H5c The Service quality of a Tax ERP is positively related to user satisfaction.

#### 3.1.6 Training & learning (TL)

Training is critical to the implementation and maintenance of a Tax ERP system. Moreover, it fosters good interactions with ERP systems (Ruivo et al., 2014). Untrained personnel has unfavorable views about an implemented ERP system (Salas and Kozlowski, 2009; Vinesh, 2014). Their study claimed that users of ERP systems tend to appreciate how simple the system is to operate since they have a complete grasp of it. Training and learning are focused on the quality of coaching provided; skill development; and the learning process. They assist users of an information system to understand the system's advantages (Shatat and Dana, 2016). Further research indicates that training and learning influence individual impact, ERP usage, and user satisfaction (Almajali et al., 2016; Costa et al., 2020). Users appreciate an ERP's simplicity of use, functionality, speed, accuracy, and flexibility after training and learning (Kabrilyants et al., 2021; Sendawula et al., 2018). We, therefore, developed the following hypotheses:

H6a Training & Learning is positively associated with individual impact.

H6b Training & Learning is positively associated with the service quality of a Tax ERP.

H6c Training & Learning is positively related to the use of a Tax ERP.

H6d Training & Learning is positively related to user satisfaction.

#### 3.1.7 Teamwork & support (TS)

Teamwork is a critical component of the workplace, requiring a group of individuals to coordinate their efforts toward achieving desired results (Moura et al., 2019). Support refers to the degree to which employees think their coworkers or ERP vendors are willing to offer them work-related help to assist them in doing their service-based responsibilities (Kurnia et al., 2019; Oh et al., 2019). The capacity of Tax ERP system users to work collaboratively is critical for effective ERP usage (Cha et al., 2015). When a Tax ERP users begin utilizing the system, teamwork and support become critical tools because they affect the service quality and frequency of use. According to Sanyal and Hisam (2018), companies should give employees the support and teams they need since they significantly contribute to enhanced performance. The following hypotheses are therefore proposed:

H7a Teamwork & support is positively related to the service quality of a Tax ERP system.

H7b Teamwork & support is positively related to the use of a Tax ERP system.

#### 3.1.8 Use (U)

The degree to which employees use an information system's capabilities is measured by the quantity of usage, the scope of use, the purpose of use, and the frequency of use. The use of a system has been proven to have a substantial impact on user satisfaction in previous research (Aditya et al., 2020; A. Aldholay et al., 2018). The individual impact component of the IS success model has also been shown to be influenced by the use of information systems (Chiu et al., 2021). According to several studies, the use of an ERP system has a substantial influence on organizational impact (Stefanovic et al., 2016; Mellouli et al., 2020). Thus we proposed the following hypotheses:

H8a The Use of a Tax ERP is positively associated with individual impact

H8b The Use of a Tax ERP is positively related to organizational impact

H8c The Use of a Tax ERP is positively related to user satisfaction

#### 3.1.9 User satisfaction (US)

This is the level of satisfaction that users have with an information system when they utilize it. There is evidence of a significant relationship between user satisfaction and individual impact (Fan and Fang, 2006; Irawan and Syah, 2017; Petter et al., 2008; Angelina et al., 2019; Salim et al., 2021). Individual productivity, job effectiveness, task performance, and job simplification may all improve if an IS user is satisfied with the system in place. Thus, we propose that:

H9 User satisfaction is positively associated with individual impact.

## 4 Method and design

A mixed-method case study (MM-CS) was used in this study. According to Guetterman and Fetters (2018), a mixed-method case study uses a nested case study for the qualitative component. The Ghana Revenue Authority (GRA) offices were studied quantitatively and qualitatively in three regions. The primary goal of the GRA is to ensure that all relevant tax laws are obeyed to provide a continuous stream of revenue for the Ghanaian government, as well as trade facilitation and the regulated and secure flow of goods across the country's borders. As a result, they have implemented a new ERP system to achieve the desired goals. The three regions selected for this study were identified because they had a large number of people working there at the time, and their units were in charge of operating the implemented tax module. The study developed the research model's design approach by demonstrating how employees use the enterprise resource planning (ERP) system. By surveying 600 users, we utilized a quantitative approach. Due to privacy considerations, the modified ERP system is referred to as ERPx in this study.

### 4.1 Measures of the constructs

To assess the success of the Tax ERP system, we utilized ten (10) constructs. The constructs and items used in the study were modified from prior research to guarantee validity. Three constructions are categorized as organizational climate values (training & learning, teamwork & support, and role clarity) among the ten (10) constructs (Furnham and Goodstein 1997; Patterson et al., 2004). Information quality (IQ), service quality (SerQ), system quality (SQ), use (U), and user satisfaction (US) are the remaining constructs (Petter et al., 2008). Individual impact and organizational impact were used to determine the net benefit.

The items used to measure the ten (10) structures are reported in Table 1. Strongly agree (7), agree (6), slightly agree (5), neither agree nor disagree (4), slightly disagree (3), disagree (2), and strongly disagree (1) were utilized on a 7-point Likert scale. The research included five items to assess information quality, four items to assess system quality, and four items to assess user satisfaction. Based on prior research, two items were attributed to the service quality and use constructs. Individual impact and organizational impact were both assessed using a five-item scale (see Table 1).

**Table 1:** Model dimensions.

Constructs	Dimension	Authors
Information Quality	Availability, relevance, timeliness, security, reliability	(Iivari, 2005; Rainer and Watson, 1995)
Service Quality	Support, assurance	(Pitt et al., 1995; Chang and King, 2005)
System Quality	Ease of use, system features, speed, accuracy, flexibility	(Gable et al., 2008; Iivari, 2005; McKinney et al., 2002)
Use	Daily use, Frequency of use	(Davis, 1993; DeLone and McLean, 2002, 2003; Petter et al., 2008)
User Satisfaction	Effectiveness, satisfaction, flexibility, adequate support	(Sirsat and Sirsat, 2016; Seddon et al., 1994; Seddon and Kiew, 1996)
Net Benefit (Individual/organizational)	Individual productivity, job effectiveness, task performance, job simplification, overall success, quality improvement	(Sedera and Gable, 2004; Gable et al., 2008; Almutairi and Subramanian, 2005)
Role clarity	Clear goals, responsibilities, use of experience	(Furnham and Goodstein, 1997; Wang et al., 2016; Curnin et al., 2015; Lau, 2015)
Training & Learning	Training for development, training quality, company learning	(Furnham and Goodstein, 1997; Hanaysha and Tahir, 2016; Islam et al., 2015)
Teamwork & Support	Collaboration, support, pressure	(Furnham and Goodstein, 1997; Hanaysha and Tahir, 2016; Caesens et al., 2016)

The research included five items to assess information quality, four items to assess system quality, and five items to assess user satisfaction. Based on prior research, two items were attributed to the service quality and use constructs (see Table 1).

The exploratory qualitative research conducted in the three (3) regions revealed that there has been a change in climate variables among workers over time. As a result, the defined organizational climate values must be included in the process of measuring the success of the ERP implementation at the GRA. Four items (training & learning, teamwork & support) and five items (role clarity) were used to assess the organizational climate constructs.

### 4.2 Data collection procedure

To evaluate the research model, data was gathered via interviews, observation, focus groups, and questionnaires. Two main deputy administrators who have been in charge of the ERP implementation from the inception took part in the interview to get a deeper knowledge of the ERPx and to help conceptualize the research model. The open-ended interview questions addressed problems that led to the adapted model and modification. The conduct of the workers was better understood via direct observation (users). It took thirty days to complete the observation process. The respondent moderator (focus group) method was utilized to collect information on the ERPx usage, function, limitations, and benefits.

The proposed research model was put to the test using survey data. The survey included the seven success dimensions developed by DeLone and McLean (1992, 2003) and Petter et al. (2008). Examples of system quality questions are "Using ERPx is easy for me" and "ERPx speeds up my work processes," while information quality questions include "ERPx enables me to safely access information" and "ERPx provides me with timely information." Additionally, service quality was assessed using example questions such as "I always get support for ERPx when necessary," and the use construct was assessed using sample questions such as "I frequently use ERPx for my work." Also, user satisfaction was assessed using example questions such as "Using ERPx enhances the quality of my work life" and "My experience with ERPx is satisfactory." The net benefit construct was also assessed using example questions such as "ERPx enhances my job efficiency" and "ERPx contributes to the overall success of GRA."

Furnham and Goodstein (1997) and Schneider et al. (1996, 2002) study were also used to modify the organizational climate variables. The degree of role clarity was determined using example questions such as "I am aware of my duties with ERPx" and "I am clear about my job priorities." Teamwork & support questions included "My department works effectively with other departments via the usage of ERPx" and "I obtained the training I required to use ERPx" and "The training I get is of high quality."

ERPx users' demographics were captured in the study. The survey could well be completed in 5 min or less by the respondents. The survey questionnaire was evaluated by four researchers and 10 ERPx users from each of the three regions for comprehension, redundancy, and clarity. The questionnaire was given to ERPx users who had been using the implemented system in GRA offices in the three regions between September 2020 and January 2021 in person. From the 600 questionnaires distributed, 555 were returned, resulting in a response rate of 92.5%. Following the COVID-19 recommendations, we utilized the accidental (convenient) sampling technique. Individual respondents were questioned whenever available. Prejudice is avoided using this method. Before distributing the questionnaire to ERPx users, we obtain authorization from management. When the independent and dependent variables are both captured by the same response technique, common method bias may emerge (Kock et al., 2021). Response bias tests, as suggested by Hulland et al. (2018), are required to verify that the sample is representative. By comparing the mean values of survey items from the first 20% of responses to the mean values of variables from the final 20% of responses, we were able to verify whether response bias had an impact on our findings. There was no statistically significant difference, indicating that there was no response bias from the single-sourced data collected for the study. In addition, the inner variance inflation factor (VIF) for each indicator in the current study was less than 5, fitting within the tolerance threshold of less than 3 (Kock, 2015, 2017). Despite the fact that the data was gathered from a single source, the study addressed common method bias.

### 4.3 Data analysis

Partial least squares structural equation modeling was used to examine the data. When the independent variables are to evaluate the number of experimental observations, partial least square (PLS) provides understandable and durable equations (Hair et al., 2019). We used PLS because when independent variables are correlated rather than orthogonal, it predicts more correctly and consistently (Leguina, 2015). As a result, the PLS allowed us to examine the relationship between organizational climate constructs (teamwork and support, training and learning, and role clarity), system quality, information quality, service quality, use, user satisfaction, individual impact, and organizational impact. We utilized SmartPLS 3.2.8 and SPSS version 23. This is done in two stages: first, a measurement model is generated, and then a structural model is developed (Hair et al., 2020). The model's PLSpredict power was also assessed.

## 5 Analysis and results

### 5.1 Demographic profile of respondents

The demographic profile of the respondents is presented in Table 2.

**Table 2:** Sample characteristics (n = 555).

Demographic variable	Category	Frequency	Percentage (%)
Age	20–24 years old	18	3.2
	25–29 years old	166	29.9
	30–34 years old	143	25.8
	35 years old and above	228	41.1
Gender	Male	282	50.8
	Female	273	49.2
ICT usage	0–5 years	57	10.3
	6–10 years	266	47.9
	above 10 years	232	41.8
Taxpayer Segment	Large Taxpayer	170	30.6
	Medium Taxpayer	240	43.2
	Small Taxpayer	145	26.1
Department	Audit	181	32.6
	Compliance	124	22.3
	Enforcement & Debt Management	114	20.5
	Accounts and Taxpayer Services	118	21.3
	Central Filling	18	3.2
Region	Central Region	145	26.1
	Ashanti Region	117	21.1
	Greater- Accra Region (Tema)	123	22.2
	Greater- Accra Region (Accra)	170	30.6

### 5.2 The measurement model

In measuring the model (outer), the indicators used are reliability, convergent validity, and discriminant validity. The item loadings were above 0.5, indicating a significant level of reliability. All the values for the composite reliability (CR) and Cronbach's alpha (α) were higher than 0.7, as shown in Table 3. The values for the AVE were also above 0.5. The figures attained from the reliability, and convergent validity test suggests that the construct variables and indicators used in the study are valid and reliable for the testing of the structural model. Table 4 shows the heterotrait-monotrait ratio (HTMT). The HTMT shows an estimate of what the actual correlation between two constructs would be if they are correctly measured.

**Table 3:** Measurement model results.

Construct	Items	VIF	Loadings^a^	AVE^b^	CR^c^	Rho_ A^d^
System Quality (SQ)	SQ1	2.327	0.845	0.760	0.927	0.902
	SQ2	2.544	0.868			
	SQ3	2.596	0.877			
	SQ4	2.846	0.898			
Information Quality (IQ)	IQ1	1.923	0.816	0.638	0.898	0.879
	IQ2	2.147	0.842			
	IQ3	1.895	0.753			
	IQ4	2.236	0.851			
	IQ5	1.840	0.723			
Service Quality (SerQ)	SerQ1	1.416	0.885	0.771	0.871	0.704
	SerQ2	1.426	0.871			
Role Clarity (RC)	RC1	2.971	0.881	0.729	0.931	0.908
	RC2	2.804	0.863			
	RC3	2.073	0.819			
	RC4	2.537	0.855			
	RC5	2.431	0.849			
Teamwork & Support (TS)	TS1	2.352	0.865	0.757	0.926	0.896
	TS2	2.293	0.853			
	TS3	2.514	0.877			
	TS4	2.544	0.886			
Training & Learning (TL)	TL1	2.742	0.889	0.769	0.930	0.901
	TL2	2.582	0.880			
	TL3	2.486	0.867			
	TL4	2.326	0.870			
Use (U)	U1	2.519	0.943	0.888	0.941	0.874
	U2	2.527	0.942			
User Satisfaction (US)	US1	2.384	0.871	0.785	0.936	0.910
	US2	2.996	0.898			
	US3	2.805	0.884			
	US4	2.953	0.891			
Individual Impact (INB)	INB1	1.763	0.878	0.730	0.890	0.869
	INB2	1.856	0.780			
	INB3	2.520	0.901			
Organizational Impact(ONB)	ONB1	1.516	0.879	0.651	0.848	0.804
	ONB2	1.463	0.801			
	ONB3	1.440	0.734			

a All Item loadings >0.5 indicate indicator reliability.

b All Average Variance Extracted (AVE) > 0.5 as indicates Convergent Reliability.

c All Composite reliability (CR) > 0.7 indicates internal Consistency.

d All Cronbach's alpha>0.7 indicates indicator Reliability.

**Table 4:** Discriminant validity (HTHT).

	INB	IQ	ONB	RC	SQ	SerQ	TL	TS	U	US
INB										
IQ	0.807									
ONB	0.134	0.106								
RC	0.842	0.792	0.139							
SQ	0.795	0.754	0.158	0.821						
SerQ	0.364	0.270	0.060	0.287	0.268					
TL	0.505	0.272	0.111	0.465	0.437	0.349				
TS	0.295	0.218	0.076	0.307	0.222	0.235	0.264			
U	0.801	0.781	0.187	0.784	0.743	0.423	0.419	0.280		
US	0.404	0.376	0.084	0.469	0.404	0.232	0.603	0.189	0.504	

Note: INB: Individual impact; ONB: Organizational impact; IQ: Information quality; SQ: System quality; SerQ: Service quality; U: Use; US: User satisfaction; RC: Role clarity; TL: Training & Learning; TS: Teamwork & Support.

### 5.3 Measurement of structural model (inner)

In measuring the structural model proposed for the study, we considered the significance of the path coefficient (hypotheses), the level of the R^2^ values, and the predictive relevance Q^2^. Bootstrapping and the PLS algorithm was used to assess the quality of the structural model. Bootstrapping is a resampling method that uses a large number of subsamples from the original dataset. The significance of the path within the structural model was determined using 5000 subsamples for this study. In order to assess the model's fitness, we used the standardized root mean square residual (SRMR) and root mean square residual covariance (rms Theta) (Hair et al., 2020). The SRMR was 0.067, and the rms Theta was 0.128. The obtained value of 0.067 shows a good fit for the model. The model's rms Theta value of 0.128 indicates that it is well-fitting.

**Figure 4:**
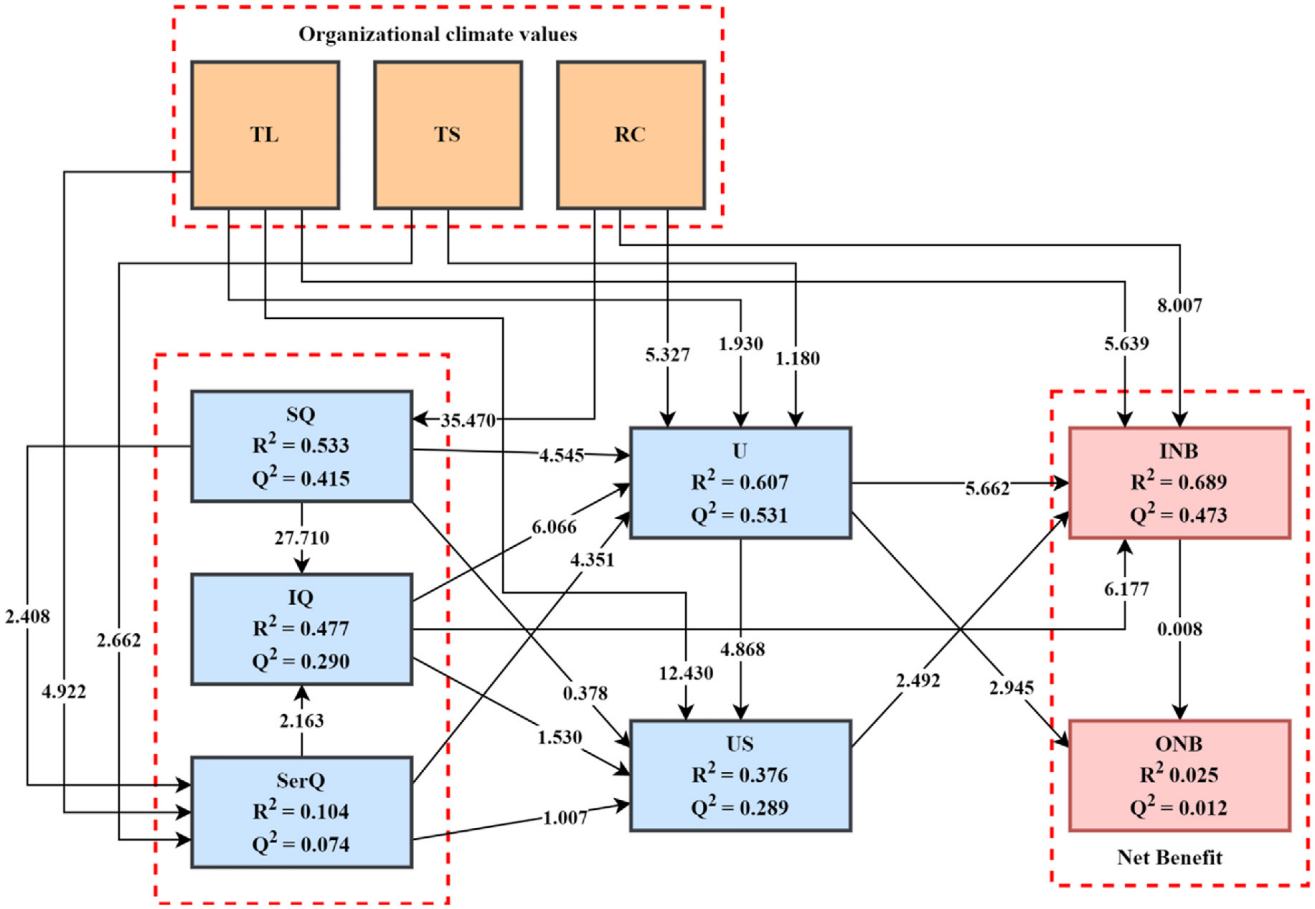
Structural model results. Organizational climate values: Training & Learning (TL); Teamwork & Support (TS); Role Clarity (RC). DeLone and McLean Model: System Quality (SQ); Information Quality (IQ); Service Quality (SerQ); Use (U); User Satisfaction (US); Individual Impact (INB); Organizational Impact (ONB).

The proposed structural model's results are shown in Figure 4. Furthermore, *service quality* and *system quality* are accountable for 47.7% of the variance in *information quality*. In addition, *system quality, training & learning, and teamwork & support* all account for 10.4% of *service quality* variance. 53.3% of the variance in *system quality* was explained by *role clarity*. *System quality, service quality, information quality, use, and training & learning* were found to account for 37.6% of the variance in *user satisfaction*. *System quality, service quality, information quality, role clarity, teamwork & support, and training & learning* were also shown to account for 60.7 % of ERPx *use*. 68.9% of the variance in i*ndividual impact* is explained by *user satisfaction, use, information quality, role clarity, and training & learning*, while 2.5% of the variance in *organizational impact* is explained by *use* and *individual impact*.

#### 5.3.1 Hypotheses testing

Table 5 shows the standardized coefficient of the pathways in the model after the constructs' reliability, convergent validity, and discriminant validity have been established. To determine the path coefficient using SmartPLS, 5000 samples were generated using a bootstrap sampling approach. As a rule of thumb, Hair et al. (2021) recommended using 5,000 bootstrap samples. The variance inflation factor (VIF) of the variables was also tested, and the findings show no issues with multicollinearity. The relationships were examined using the standardized beta, t-values, and p-values. Individual impact does not influence organizational impact statistically (β = 0.001, p = 0.990), resulting in H1 rejection. Individual impact (β = 0.267, p = 0.000) and use (β = 0.328, p = 0.000) were both influenced by information quality, confirming H2a-H2b. H2c, on the other hand, was rejected because user satisfaction is not statistically influenced by information quality (β = 0.074, p = 0.130). Information quality (β = 0.672, p = 0.000), service quality (β = 0.112, p = 0.020), and use (β = 0.184, p = 0.000) all exhibit statistical significance with system quality. As a result, H4a-c was accepted. Because system quality does not statistically influence user satisfaction (β = -0.021, p = 0.710), H4d was rejected. Information quality (β = 0.074, p = 0.030) and use (β = 0.138, p = 0.000) are both influenced by service quality. As a result, H5a-b was accepted. H5c, on the other hand, was rejected because user satisfaction is not statistically influenced by service quality (β = -0.037, p = 0.310). H8a-c was also accepted since it has a statistically significant positive influence on the individual impact (β = 0.233, p = 0.000), organizational impact (β = 0.163, p = 0.000), and user satisfaction (β = 0.256, p = 0.000). H9 was rejected because it shows that user satisfaction has a negative and significant influence on individual impact (β = -0.077, p = 0.010). Individual impact (β = 0.370, p = 0.000), system quality (β = 0.745, p = 0.000), and usage (β = 0.259, p = 0.000) are statistically associated with role clarity. As a result, H3a-c was approved. Individual impact (β = 0.175, p = 0.000), service quality (β = 0.209, p = 0.000), and user satisfaction (β = 0.454, p = 0.000) are also statistically influenced by training and learning. Hence, H6a-b and H6d were accepted. However, training and learning had no impact on use (β = 0.058, p = 0.050). As a result, H6c is rejected. Finally, teamwork and support have a significant impact on service quality (β = 0.116, p = 0.010). Thus, H7a is accepted. H7b was rejected because teamwork and support had no impact on use (β = 0.033, p = 0.240).

**Table 5:** Hypotheses results.

Hypotheses	Relationship	Std. Beta	Std. Error	t-value	p-values	Decision	95% CI LL	95% CI UL
H1	INB - > ONB	0.001	0.064	0.008	0.990	Rejected	-0.120	0.120
H2a	IQ - > INB	0.267	0.043	6.177	0.000	Accepted	0.182	0.350
H2b	IQ - > U	0.328	0.054	6.066	0.000	Accepted	0.223	0.430
H2c	IQ - > US	0.074	0.048	1.530	0.130	Rejected	-0.020	0.170
H3a	RC - > INB	0.370	0.046	8.007	0.000	Accepted	0.282	0.460
H3b	RC - > SQ	0.745	0.021	35.470	0.000	Accepted	0.702	0.790
H3c	RC - > U	0.259	0.049	5.327	0.000	Accepted	0.162	0.350
H4a	SQ - > IQ	0.672	0.024	27.710	0.000	Accepted	0.622	0.720
H4b	SQ - > SerQ	0.112	0.046	2.408	0.020	Accepted	0.017	0.200
H4c	SQ - > U	0.184	0.040	4.545	0.000	Accepted	0.108	0.260
H4d	SQ - > US	-0.021	0.052	0.378	0.710	Rejected	-0.120	0.080
H5a	SerQ - > IQ	0.074	0.034	2.163	0.030	Accepted	0.006	0.140
H5b	SerQ - > U	0.138	0.032	4.351	0.000	Accepted	0.075	0.200
H5c	SerQ - > US	-0.037	0.037	1.007	0.310	Rejected	-0.110	0.040
H6a	TL - > INB	0.175	0.031	5.639	0.000	Accepted	0.114	0.240
H6b	TL - > SerQ	0.209	0.042	4.922	0.000	Accepted	0.126	0.290
H6c	TL - > U	0.058	0.030	1.930	0.050	Rejected	-0.000	0.120
H6d	TL - > US	0.454	0.036	12.430	0.000	Accepted	0.383	0.530
H7a	TS - > SerQ	0.116	0.044	2.662	0.010	Accepted	0.030	0.200
H7b	TS - > U	0.033	0.028	1.180	0.240	Rejected	-0.020	0.090
H8a	U - > INB	0.233	0.041	5.662	0.000	Accepted	0.155	0.320
H8b	U - > ONB	0.163	0.054	2.945	0.000	Accepted	0.051	0.260
H8c	U - > US	0.256	0.053	4.868	0.000	Accepted	0.153	0.360
H9	US - > INB	-0.077	0.031	2.492	0.010	Rejected	-0.140	-0.000

p < 0.05; p < 0.01; p < 0.001 ∗CI LL: confidence interval lower ∗CI UP: confidence interval upper R^2^ = (INB = 0.689; ONB = 0.025; U = 0.607; US = 0.376; SQ = 0.533; SerQ = 0.104; IQ = 0.477); Q^2^ = (INB = 0.473; ONB = 0.012; IQ = 0.290; SQ = 0.415; SerQ = 0.074; U = 0.531; US = 0.289). Effect size impact indicator is according to Cohen (1988), f^2^ values: 0.35 (large), 0.15 (medium), and 0.02 (small). Predictive relevance (q^2^) of predictor exogenous latent variables as according to Henseler et al. (2009), q^2^ values: 0.35 (large), 0.15 (medium), and 0.02 (small).

#### 5.3.2 PLSpredict analysis

Our proposed model's out-of-sample predictive power was also examined. The PLSpredict model is utilized with 10 folds and one repetition to mimic how the PLS model would eventually be used to predict a new observation, rather than utilizing the average across multiple models (Shmueli et al., 2016). We focus our study on the proposed model's key target construct Net benefit (Table 6), but we also show the prediction statistics of all the other endogenous constructs' indicators to exemplify the interpretation.

**Table 6:** PLSpredict assessment of variables.

Item	PLS-SEM		LM	PLS-SEM -LM
	RMSE	Q^2^_predict_	RMSE	RMSE
INB1	0.840	0.666	0.752	0.088
INB2	1.177	0.170	1.176	0.001
INB3	1.293	0.384	1.280	0.013
IQ1	1.368	0.400	1.282	0.086
IQ2	1.379	0.376	1.329	0.050
IQ3	1.355	0.172	1.351	0.004
IQ4	1.389	0.345	1.367	0.022
IQ5	1.270	0.172	1.274	-0.004
ONB1	1.260	0.008	1.282	-0.022
ONB2	1.165	0.007	1.174	-0.009
ONB3	1.235	0.008	1.258	-0.023
SQ1	1.353	0.345	1.350	0.003
SQ2	1.366	0.383	1.366	0.000
SQ3	1.217	0.418	1.228	-0.011
SQ4	1.084	0.514	1.096	-0.012
SerQ1	1.702	0.080	1.691	0.011
SerQ2	1.683	0.063	1.674	0.009
U1	1.270	0.418	1.275	-0.005
U2	1.285	0.445	1.281	0.004
US1	1.506	0.332	1.492	0.014
US2	1.457	0.258	1.475	-0.018
US3	1.451	0.234	1.452	-0.001
US4	1.581	0.233	1.590	-0.009

The results show that the indicators yielded values for Q^2^_predict_ that are greater than 0. When the RMSE values from the PLS-SEM analysis are compared to the naïve LM benchmark (see Table 6), the PLS-SEM analysis yields fewer prediction errors for the vast majority of its indicators. When using PLS-SEM to estimate the model, the RMSE values for indicators IQ5, ONB1, ONB2, and ONB3 are 1.270, 1.260, 1.165, and 1.235, respectively, but the RMSE values for the naïve LM are 1.274, 1.282, 1.174, and 1.258. The RMSE value for indicator SQ2 was 1.366, which was the same as the RMSE value for the naïve LM. The two prediction error distributions should, in general, overlap closely, according to Danks and Ray (2018). The RMSE values for the indicators SQ3, SQ4, U1, US2, US3, and US4 are 1.217, 1.084, 1.270, 1.457, 1.451, and 1.581, respectively, whereas the RMSE values for the LM are 1.228, 1.096, 1.275, 1.475, 1.452, and 1.590. While it is true that some of the RSME values for the naïve LM benchmark were less than the PLS-SEM, the difference was not high. As shown in the PLS-SEM indicators, RMSE values for INB2, IQ3, SQ1, and SerQ1 were 1.777, 1.355, 1.353, and 1.702, respectively, whereas RMSE values for naïve LM were 1.176, 1.351, 1.350, and 1.961.

According to Shmueli et al. (2019), the thumb rule for running PLSpredict for PLS-SEM less than LM for the majority of the indicators is: When the majority (or the same number) of indicators in the PLS-SEM analysis produce smaller prediction errors than the LM, the predictive power is considered medium. As a result, the data in Table 6 shows that the PLS-SEM model used in this analysis has a medium predictive power.

## 6 Discussion

The current study aimed to propose an ERP success model that takes into account organizational climate values and can be used to assess the success of an ERP implemented in a developing country. We examined the variables that influence the use of an ERP system, user satisfaction, information quality, service quality, and system quality. Although some hypotheses were rejected, the majority of hypotheses empirically support the assessment of a revenue collection ERP system success in a developing nation, as shown in Table 5. The DeLone and McLean IS success model's evaluation of an implemented ERP system in a revenue company was significantly influenced by the proposed organizational climate values, according to the model's result.

The findings of the study show that understanding one's roles and responsibilities in an ERP system improves productivity, work effectiveness, task performance, and simplifies one's job. This demonstrates that the clarity of one's role has a significant influence on individual impact. This is in line with the findings of the Pahi et al. (2020) study. It also had an impact on the system quality of the ERP system and how it was used. This also means that ERP users who are aware of their role within an organization and the ERP system they are using will enjoy the system's ease of use, which includes functionality, speed, accuracy, and flexibility. The administrators acknowledged such, which further verifies the outcomes of the interview that;

*“You know, before we agreed on the system's deployment, we were able to divide taxpayers into three categories: large taxpayers, medium taxpayers, and small taxpayers, all of whom were handled by their various taxpayer offices around the country. As a result, each unit's role was clearly defined to ensure that staffs were aware of what was expected of them both before and after the implementation. Since 17 modules of the Tax Administration System are to be handled by the Domestic Tax Revenue Division, which happens to be our unit, I must state that the staffs have demonstrated that they understand their roles since the system's inception”* (Administrator 1)

*“I feel that most of the employees have mastered their role with the system over time, and while it is too early to say, I believe it has helped them appreciate the system's features and discovered that it is simple to use with time. Also, if you ask about speed and flexibility, I must admit that owing to a few internet connectivity difficulties, the system was originally slow to operate, but as the employees acquire expertise, the system has become more adaptable. With this system in place, we anticipate increased success”* (Administrator 2)

Role clarity also increases employees' use of the ERP system, particularly if it is mandatory. This finding was anticipated since it is in line with prior studies (Kalliath et al., 2012; Orgambdez and Almeida, 2020). The study's interview findings back up this conclusion, as the administrators' state:

*“It's too early to say if the employees' awareness of their role in the system will lead to their usage of the system. This is because, over time, this system will become mandatory for all units of this company, as the government seeks to assist us in improving our customer service while also reducing the strain that frequently builds inside this organization. I understand that once the system is mandated, it will be used continuously. However, it is still necessary to assist employees in understanding their roles in the system so that they do not end up undermining the system's continued usage or the benefits that come with it”* (Administrator 2)

Training and learning are critical elements of measuring an ERP system's success since they improve users' satisfaction with the system, the service quality outcome, and even have an individual impact, as evidenced in the findings. This shows that when training and learning are effectively conducted inside an organization that needs ERP system users to use it, the users are generally satisfied. Users' productivity, effectiveness, job performance, and work simplicity are all favorably impacted by the quality of coaching provided, skill development, and the learning process. Almajali et al. (2016) and Costa et al. (2020) found similar results. The findings of the interviews also show that:

*“In terms of training and learning, I believe these are the most important components that might contribute to the system's success. As a result, I would say that management has ensured that even before the system was implemented, they helped build capacity in computer literacy, IT infrastructure Library (ITIL), and that after the system was implemented; staff members were given user acceptance training in both the e-Tax administration system and the e-Business registration system. This, I believe, will go a long way toward allowing system users to be satisfied with the system and not feel stressed out by the technology. Because the advent of new technology brings with it its own plethora of challenges”* (Administrator 1)

The amount of support and assurance users get from both internal and external IT technicians is also influenced by training and learning. This is due to their ability to explain the particular challenges they face and, when led through them, overcome the problem. This is to guarantee that, as indicated by the study's findings, users are satisfied with the system. This result is likewise in line with Shatat and Dana (2016) findings. In contrast to Almajali et al. (2016), our findings show that ERP users' propensity to use an ERP is undeterred by training and learning, even when the implemented system is mandatory. In the interview, the administrator also stated:

*“I must mention that the training and learning sections presented throughout this time have helped users of the system recognize who to contact when they have a problem and how to report those problems”* (Administrator 1)

Teamwork and support had a significant positive impact on service quality, but they had a negative impact on ERP use. This shows how internal ERP users appreciate the system's support and trust when it provides appropriate assistance and teamwork. This study is comparable to that of Kurnia et al. (2019) and Oh et al. (2019). This echoes the administrator's views.

*“Teamwork and support are two things we've pushed for. We believe that if the departments that use the system can form a strong team, they will be able to overcome any future obstacles. I should also mention that the organization received continuous help from the vendor and internal IT unit experts during the first implementation phase of the system. However, with time, it becomes more difficult to enlist the team's assistance in resolving an issue. Because we occasionally need to schedule a flight for technicians to come over and fix a problem. At the very least, with solid collaboration, as you recommended, I believe the system's service quality will be excellent”* (Administrator 2)

However, according to the findings of this study, ERP users' cooperation, support, and pressure have no significant influence on how they use the system. This is in direct contrast to prior studies (Cha et al., 2015). The study's conclusions, on the other hand, were not accepted by the administrators.

*“Also, I believe that with the appropriate teamwork and support, the pace at which system users utilize the system will increase, and that continuous utilization will not be an issue”* (Administrator 2).

Each of the three quality constructs assessed (information quality, service quality, and system quality) had a positive impact on users' willingness to use an ERP. This shows that for internal ERP users to benefit from an IS system, the information generated by the system must always be accessible, relevant, timely, secure, and reliable. Support and assurance problems should be addressed while meeting the service quality need for workers to use an ERP. This finding is in line with previous research since it enables ERP users to learn about the system's ease of use, features, speed, accuracy, and adaptability (Aldholay et al., 2019; Chen, 2020; Stefanovic et al., 2016; Mishra and Geleta, 2020; Kumar et al., 2020; Nishant et al., 2019). Regardless, the three quality constructs of the IS success model applied in this study had a negative impact on user satisfaction. However, just because an ERP system has the qualities embodied in these three information systems (IS) quality constructs: availability, relevance, timeliness; security; reliability; support; assurance; ease of use; system features; speed; accuracy; and flexibility; does not mean ERP users will be completely satisfied with the system. These results are incongruent with prior studies analyzed in the present study (Alkraiji, 2020; Sachan et al., 2018; Veeramootoo et al., 2018; Al-Okaily et al., 2021; Arain et al., 2019; Mohammadi, 2015; Wang and Teo, 2020). The results are supported by the results of the interviews.

*“Talking about the quality of the system in terms of information quality, service quality, and system quality, these were the key priorities taken into account in selecting the system. This was because, if the system's expected attributes are not in excellent shape, putting it to use by employees becomes difficult. However, I must state that the existing system exemplifies wonderful traits. As we may access information from anywhere, seven days a week, 24 hours a day, it enables system users to utilize the system frequently and every day. If you ask me if these qualities affect the satisfaction of system users, I would say that for the time being, the mandatory nature of the system makes it difficult to determine whether all users are satisfied with the system, even if they exhibit the highest levels of information quality, system quality, and service quality”* (Administrator 1)

When the interrelationships between the quality constructs were examined, it was discovered that service quality and system quality had a significant impact on information quality. Indicating that an implemented ERP system that is easy to use, has flexible system features, is fast, accurate, and flexible, and provides the necessary support and assurance when needed has a significant impact on information quality attributes such as availability, relevance, timeliness, security, and dependability, among other attributes. This study is similar to Santa et al. (2019) findings. The interview findings corroborate these conclusions.

*“Yes, I must add that the assistance and assurance we have received so far from the system's vendors, as well as the ease with which workers can use the system and the user-friendly nature of its features, have contributed significantly to an improvement in the quality of information we generate currently for future organizational initiatives. We are able to obtain information relating to imports in order to make decisions and administer taxes”* (Administrator 1)

In addition, system quality has been shown to have a significant impact on service quality. This signifies that an ERP system's attributes, such as ease of use, system features and speed, accuracy, and flexibility, have an impact on the service providers' and internal IT technicians' support and assurance. The findings of this study are consistent with those of Ameen et al. (2019) and Mukamurenzi et al. (2019).

The ERP system's information quality was also found to have a significant influence on individual impact. Individual impact, on the other hand, has no significant influence on organizational impact. This suggests that an ERP system with information quality features like availability, relevance, timeliness, security, and dependability has a significant impact on the user's productivity, task performance, and job simplicity. This result is similar to Chen et al. (2019) findings. Individual impact outputs such as increased productivity, work effectiveness, task performance, and job simplicity, on the other hand, do not influence an organization's overall success and quality improvement. These findings conflict with the research findings of Roky and Meriouh (2015) and Krasniqi et al. (2019). The interview findings confirm that the quality of information has a significant influence on individual impact.

*“I must state that the higher-quality information we get from the existing system is frequently prompt, secure, and dependable. Unlike the traditional system, which allowed staff easy access to sensitive information, the present system requires several levels of passwords to identify what the information is used for. As a result, users of the system become more effective and efficient, since if we can e-file and e-pay, we can link to the banks, so that if someone makes a quick payment, it can easily reach us, bringing reconciliation efficiency”* (Administrator 2)

Individual impact, on the other hand, has no significant impact on organizational impact, according to the interview data.

*“Although most employees have become more productive, efficient, and comfortable performing tasks assigned to them as a result of the system's use over time, I won't say it directly accounts for our company's overall success, even though it has improved the quality of work done internally within our organization”* (Administrator 2)

The use of an ERP system has a significant influence on user satisfaction, individual impact, and organizational impact, according to this study. Indicating that users are satisfied with an ERP system's efficiency, satisfactoriness, adaptability, and enough support since they use it on a daily or frequent basis. Users enhance their productivity and effectively perform their responsibilities as a result of daily or frequent usage, as well as the simplicity of how they do their work, overall organizational success, and quality improvement. This is in line with the results of a recent study (Aditya et al., 2020; Mellouli et al., 2020). According to the findings of this study, user satisfaction has a negative influence on individual impact, although the two have a strong correlation. This result contradicts Angelina et al. (2019) and Salim et al. (2021) findings that information systems make users' lives easier, more cost-effective, time-efficient, productive, and effective. Thus, while users of an information system may be satisfied with its effectiveness, flexibility, and adequate assistance, it has a negative influence on their productivity, work simplicity, and task performance.

Finally, service quality and system quality account for 47.7% of the variance in information quality, according to the study. System quality, training & learning, and teamwork & support account for 10.4% of service quality variance. Role clarity accounted for 53.3% of the variance in system quality. System quality, service quality, information quality, use, and training & learning all contributed to 37.6% of user satisfaction. Furthermore, system quality, service quality, information quality, role clarity, teamwork & support, and training & learning accounted for 60.7% of ERP use. The variance (68.9%) in individual impact is accounted for by user satisfaction, use, information quality, role clarity, and training & learning, while the variance (2.5%) in organizational impact is accounted for by use and individual impact.

### 6.1 Theoretical implications

The present study, in particular, proposed a model for assessing the success of a tax ERP system in a developing nation. Different kinds of data collecting methods (interview, observation, and focus group) were utilized in addition to quantitative data collection; however, they were not reported in the present study. This was because they were used to help the researchers better understand the current ERPx system, the challenges it poses, and the attitudes of users toward it. The proposed model incorporates both IS success theory (DeLone and Mclean, 1992, 2003) and organizational climate characteristics (Furnham and Goodstein, 1997; Wang et al., 2016; Curnin et al., 2015; Lau, 2015; Hanaysha and Tahir, 2016; Islam et al., 2015; Caesens et al., 2016).

According to the findings of this study, a more in-depth evaluation of organizational climate values (training and learning, teamwork and support, and role clarity) when measuring the success of a tax ERP system in a developing country contributes significantly to the ERP system's success measurement.

Furthermore, the results show that system quality, service quality, information quality, role clarity, training & learning, and teamwork & support all play a key part in determining the use of an ERP system. System quality, service quality, information quality, use, and training & learning all have a significant role in whether or not users are satisfied with the use of a tax ERP system, according to the current study. It was discovered that role clarity contributes to the system quality of a tax ERP system. According to the findings, two variables influence the information quality of a tax ERP system: system quality and service quality. System quality, training & learning, and teamwork & support are all factors that contribute to service quality. Use, information quality, role clarity, and training & learning all contribute to individual impact when a tax ERP is put in place. The research also discovered that organizational impact was dependent on the use of the ERP and individual impact.

### 6.2 Practical implications

This study offers governments, ERP providers, and companies in developing countries an evaluation model for assessing the success of a tax ERP system. Specifically, the findings showed that certain construct-relationships that had been modified for the study were more significant than others.

The practical implications of the study led us to conclude that for governments and organizations to use mandatory tax ERP systems in developing countries, top management should pay close attention to system quality, service quality, information quality, role clarity, teamwork & support, training & learning, and other aspects of system operation. When adopting such systems, it is important to consider the system quality, service quality, information quality, simplicity of use, and training & learning, since all of these factors contribute to the system's overall user satisfaction.

Aside from that, for internal users of an ERP system inside a company to have an individual impact, they must first establish a habit of using the system, since they will ultimately become satisfied with it. Organizations and ERP suppliers should also take steps to ensure that the system's output (IQ) is relevant, timely, dependable, easily available, and securely protected. To achieve this, users must be informed of their roles within the deployed ERP system, and they must get comprehensive training and educational opportunities.

It is essential to have clear roles since it helps to the formulation of an ERP system quality, which includes its ease of use and flexibility as well as its accuracy, speed, and features. In order to provide better service to governments and companies that use tax ERP, ERP suppliers should prioritize system quality, training & learning, as well as teamwork & support. These constructs have been shown to enhance service quality.

In conclusion, the individual impact and successful usage of a tax ERP system should be a top concern for businesses that use them since they contribute to the overall performance and quality improvement of the company.

### 6.3 Limitations and future work

Due to the fact that the current study validated a comprehensive model for assessing the success of a tax ERP system in a developing country, it leaves out some relationships between the proposed constructs that may explain ERP success in the future. Additionally, it will be important to conduct studies that explore other construct relationships between IS quality constructs such as SerQ→ SQ, IQ→SQ, and IQ→SerQ, among others. Research on the influence of teamwork & support, as well as the clarity of the job role, on user satisfaction, should be carried out in future studies. Finally, the impact of user satisfaction with an information system on the use of a tax ERP system can be examined.

The study was also restricted to a single revenue collection agency, which happens to be the only one in Ghana, West Africa, which is allowed to collect taxes on behalf of the government. As a consequence, since this sample does not represent a larger population, care should be taken when extrapolating the results. Future research may also focus on expanding the scope of the study to include comparable tax ERP systems in other West African areas, as well as comparing them to countries in Europe and Asia.

## 7 Conclusions

ERP systems require substantial support, training, teamwork, role clarity, and learning from strategy through execution. But it also presents a huge potential to enhance tax data quality and efficiency. There is a need for an IS success model that can include these factors to help measure the success of tax ERP systems. The technological revolution has influenced tax collection, administration, and compliance in both emerging and developed nations. In addition to the DeLone and McLean IS success model, the present empirical study proposed a theoretical model that included organizational climate values such as training & learning, teamwork & support, and role clarity. The study also examined the factors that influence the use of a tax ERP system by internal users, user satisfaction, information quality, and system quality.

The proposed organizational climate and IS success model factors were hypothesized. In this study, we assessed the influence of organizational climate factors on IS use and the quality dimensions. All of these dimensions were verified, thereby validating the model. The model was tested on a tax revenue agency in three distinct areas in Ghana, West- Africa where this institution has implemented a tax ERP system. The study's findings indicate that the proposed organizational climate variables and the DeLone and McLean IS success model components effectively assess a tax ERP system's success. The key variables that influence the use of tax ERPs are system quality, service quality, information quality, role clarity, teamwork & support, and training & learning. System quality, service quality, information quality, use, and training & learning were found to be important determinants in internal tax ERP system users' satisfaction.

The tax ERP system's information quality was also attributed to service and system quality. Variables like system quality, training & learning, and teamwork & support must be addressed for substantial service quality. However, role clarity accounted for the bulk of the variation in system quality. Individual impact was measured by use, information quality, role clarity, and training & learning, whereas organizational impact was measured by tax ERP system use and individual impact. As a result of these findings, the success of a tax ERP system must be measured by factoring in the organizational climate variables such as training & learning, teamwork & support, and role clarity.

## Declarations

### Author contribution statement

Godwin Banafo Akrong; Shao Yunfei & Ebenezer Owusu: Conceived and designed the experiments; Analyzed and interpreted the data; Contributed reagents, materials, analysis tools or data, wrote the paper, performed the experiments; Wrote the paper.

### Funding statement

This research did not receive any specific grant from funding agencies in the public, commercial, or not-for-profit sectors.

### Data availability statement

The data that has been used is confidential.

### Declaration of interest’s statement

The authors declare no conflict of interest.

### Additional information

No additional information is available for this paper.

## Figures and Tables

**Table:** Table 7. Survey items for the constructs.

Construct	Numbers	Items
Role Clarity	RC.1	I have clear goals and objectives for my job.
	RC.2	With ERPx, I am clear about my job priorities.
	RC.3	I am fully aware of my duties in relation to ERPx.
	RC.4	The organization makes good use of frequent ERPx users.
	RC.5	Workers in the company are of an exceptional quality.
Teamwork & Support	TS.1	My department uses ERPx effectively with other departments.
	TS.2	My team members generally support the use of ERPx.
	TS.3	My teammates seldom put me under work pressure when utilizing ERPx.
	TS.4	The company's workforce tends to help each other out.
Training and Learning	TL.1	I have received the training I need to use ERPx.
	TL.2	In general, this company learns as much as is practically possible from its activities with ERPx.
	TL.3	The training I receive is of high quality.
	TL.4	I get the training I need to further develop my skills in using ERPx.
System Quality	SQ.1	Using ERPx is easy for me.
	SQ.2	Functions and features provided by ERPx are useful for my work.
	SQ.3	The use of ERPx speeds up my work operations.
	SQ.4	The services of ERPx are always available for my work.
Information Quality	IQ.1	ERPx ensures information availability.
	IQ.2	ERPx provides me with relevant information.
	IQ.3	ERPx guarantees the reliability of information.
	IQ.4	ERPx allows me to access information securely.
	IQ.5	ERPx generates information on time.
Service Quality	SerQ.1	There is adequate technical support from the ERPx system's provider.
	SerQ.2	The ERPx can be relied on to provide information as when needed.
Use	U.1	I use ERPx frequently because it enables me to accomplish tasks more quickly.
	U.2	I use ERPx several times a day for my work because it has improved my job performance.
User Satisfaction	US.1	I am satisfied with the functions of the ERPx.
	US.2	The ERPx has eased work processes.
	US.3	I receive adequate support when using ERPx for my work.
	US.4	I am generally satisfied using the ERPx.
Individual Impact	INB.1	The ERPx improves my performance of tasks.
	INB.2	The ERPx makes me effective.
	INB.3	The ERPx saves me time.
Organizational Impact	ONB.1	The ERPx system enhance the simplification of job.
	ONB.2	The ERPx has improved services and overall success of my department.
	ONB.3	The ERPx facilitated exchange of organizational data.
